# Evaluation of blood-count-derived inflammatory markers in patients with idiopathic epiretinal membrane

**DOI:** 10.1186/s12886-025-04047-2

**Published:** 2025-04-18

**Authors:** Serdar Bilici, Kardelen Ezgi Şahin Elarslan, Numan Küçük, Suat Hayri Uğurbaş

**Affiliations:** School of Medicine, Department of Ophthalmology, Zonguldak Bulent Ecevit University, Zonguldak, Turkey

**Keywords:** Idiopathic epiretinal membrane, Optical coherence tomography, Systemic inflammation markers

## Abstract

**Background:**

To assess the role of inflammation in the pathogenesis of idiopathic epiretinal membrane (iERM) by evaluating blood-count-derived inflammatory marker levels.

**Methods:**

The medical records of patients diagnosed with iERM and cataract patients with normal fundus examinations were analyzed retrospectively. Levels of neutrophils, monocytes, lymphocytes, and thrombocytes were obtained from blood samples. Systemic inflammatory markers, including neutrophil-lymphocyte ratio (NLR), platelet-lymphocyte ratio (PLR), systemic immune-inflammation index (SII), and systemic inflammatory response index (SIRI) were calculated and compared between the two groups. The receiver operating characteristic curve (ROC) analysis was performed to determine the best cutoff value of NLR, PLR, SII, and SIRI in iERM.

**Results:**

In total, 91 iERM cases and 95 controls were included in the study. iERM patients had significantly higher NLR (2.25 vs. 1.91, *p* = 0.003), PLR (117.22 vs. 113.33, *p* = 0.042), SII (529.45 vs. 472.57, *p* = 0.003), and SIRI (1.25 vs. 0.90, *p* < 0.001). The area under the curve of NLR, PLR, SII, and SIRI in differentiating patients with iERM and controls was 0.637, 0.608, 0.645 and 0.660, respectively, according to ROC analysis. The best cutoff values (with sensitivity and specificity) were 1.95 (60.4% and 52.6%) for NLR, 116.7 (54.9% and 55.7%) for PLR, 498.03 (58.2% and 58.9%) for SII, and 1.07 (62.6% and 64.6%) for SIRI.No significant differences in inflammatory markers were found across iERM stages.

**Conclusion:**

Patients with iERM exhibit higher levels of blood-count-derived inflammatory markers, suggesting a link between systemic subclinical inflammation and iERM development. However, these markers do not correlate with iERM severity. Further research with larger cohorts and broader inflammatory marker analysis is needed to elucidate the role of systemic inflammation in iERM pathogenesis.

## Background

Idiopathic epiretinal membrane (iERM) is a condition characterized by the formation of a thin layer of fibrous tissue on the surface of the retina [1, 2]. It’s a common condition in the elderly population that leads to visual impairment and metamorphopsia with the prevalence from 7 to 11.8% [3]. The precise mechanisms behind the development of iERM are not fully understood. The formation of an ERM involves cell migration and proliferation, the creation of an extracellular matrix, and tissue contraction. It is widely accepted that cortical remnants of the vitreous after the posterior vitreous detachment (PVD) have a significant role in the iERM pathogenesis by providing an environment where glial cells can proliferate and undergo transformation [4, 5]. Previous studies have highlighted the role of growth factors and cytokines in the formation of iERM, indicating that inflammatory mechanisms are also implicated in its development [6–9].

Blood-count-derived inflammatory markers have recently been the focus of research. These biomarkers have demonstrated associations with various systemic diseases and ocular disorders, suggesting their potential utility in identifying underlying inflammatory processes [10–13]. Understanding the relationship between systemic inflammation and retinal diseases could offer valuable insights into the pathogenesis of iERM. Consequently, the primary objective of this study was to assess the relation of blood-count-derived inflammatory markers with iERM and its stages.

## Materials and methods

The study was approved by the Ethics Committee of Zonguldak Bulent Ecevit University (approval number: 2024/07–09) and adhered to the Declaration of Helsinki’s guidelines. The Ethics Committee waived the need for written informed consent due to the retrospective nature of the research.

**Fig. 1:**
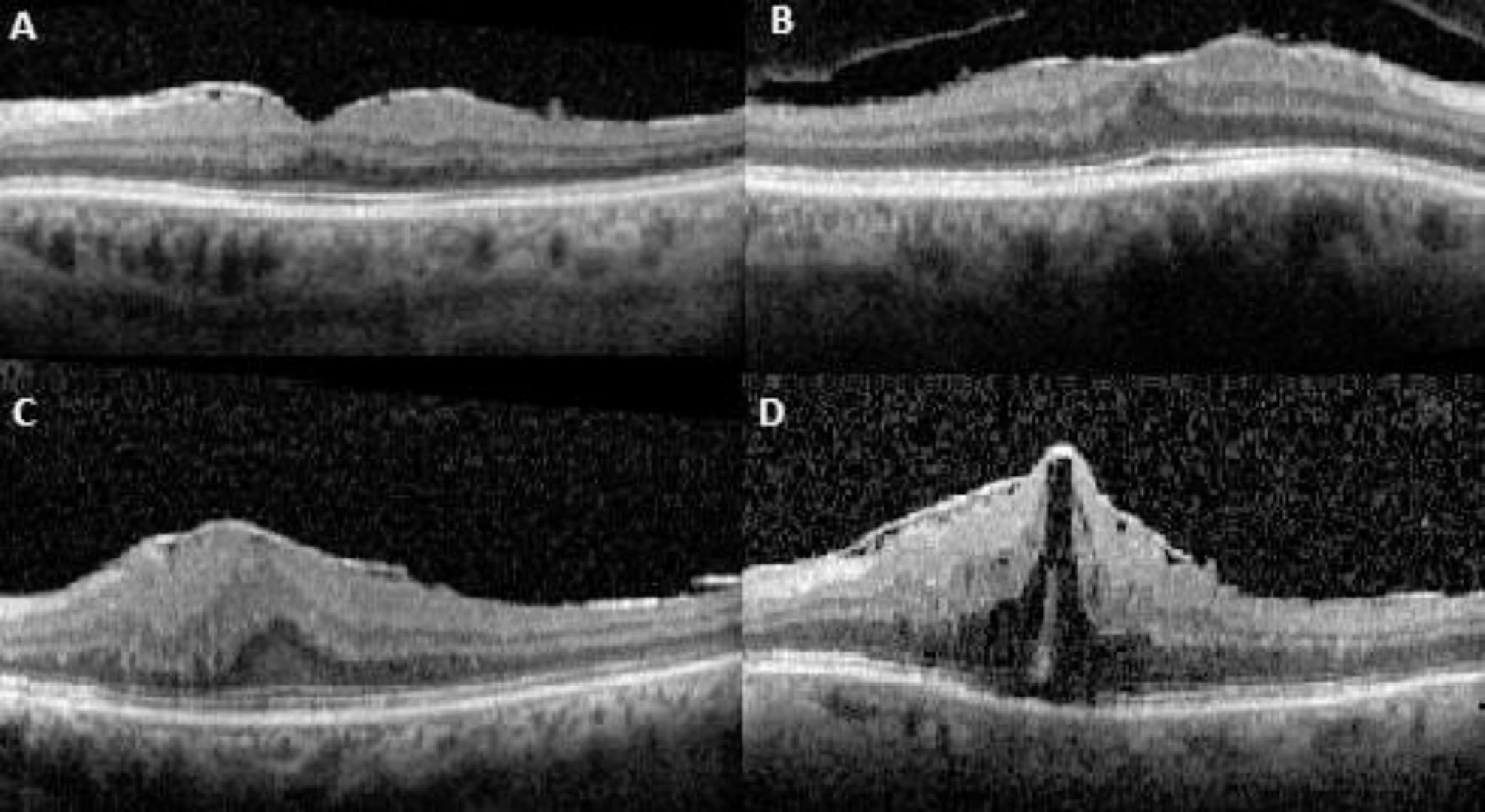
Optical Coherence Tomography Based Grading of Idiopathic Epiretinal Membrane. a. stage 1, presence of the foveal pitand well-defned retinal layers; b. stage 2, absence of the foveal pit and presence of well-defned retinal layers; c. stage 3, absence of the foveal pit and well-defned retinal layers and presence of ectopic inner foveal layer; d. stage 4, absence of the foveal pit and disrupted retinal layers and presence of ectopic inner foveal layer

The medical records of patients diagnosed with iERM at Zonguldak Bulent Ecevit University Hospital from June 2021 to March 2024 were reviewed retrospectively. Besides the comprehensive fundoscopic examinations utilizing slit-lamp biomicroscopy, spectral-domain optical coherence tomography (SD-OCT; Spectralis, Heidelberg Engineering, Germany) was used to confirm the diagnosis of iERM. In addition, fundus fluorescein angiography (FFA) was performed to exclude other conditions like vascular diseases (e.g., diabetic retinopathy, retinal vascular occlusion), inflammatory disorders like uveitis or malignancies. The severity of ERM was categorized using the Govetto classification system, which consists of four stages: Stage 1 (foveal pit present with well-defined retinal layers), Stage 2 (absence of foveal pit with well-defined retinal layers), Stage 3 (absence of foveal pit, well-defined retinal layers, and presence of an ectopic inner foveal layer), and Stage 4 (absence of foveal pit, disrupted retinal layers, and presence of an ectopic inner foveal layer) (Fig. 1) [14].

Control subjects were drawn from patients who underwent detailed fundus examinations using SD-OCT and comprehensive blood examinations as part of their preoperative evaluation for cataract surgery. Exclusion criteria included any history that could predispose to secondary ERM, such as previous intraocular surgeries, ocular trauma (blunt or penetrating), retinal detachment, uveitis, diabetic retinopathy, venous occlusion, age-related macular degeneration, high myopia (greater than 6 diopters), autoimmune disorders, cardiovascular diseases, malignancies, and the use of medications that might affect blood parameters (e.g., chemotherapeutic agents, iron supplements, corticosteroids).

Neutrophil, monocyte, lymphocyte, and thrombocyte levels were obtained from preoperative blood samples (FFA or cataract surgery). All blood samples were collected in the same hospital’s designated blood collection unit, following standard procedures: using a 21-gauge syringe and EDTA tubes, and in a non-fasting state. Systemic inflammatory biomarkers were evaluated, including the Neutrophil-to-Lymphocyte Ratio (NLR, unitless), Platelet-to-Lymphocyte Ratio (PLR, unitless), Systemic Immune-Inflammation Index (SII, 10^9^/L), calculated as (neutrophil × platelet)/lymphocyte, and Systemic Inflammatory Response Index (SIRI, 10^9^/L), calculated as (neutrophil × monocyte)/lymphocyte [15].

### Statistical analysis

The data analysis was performed using IBM SPSS version 22.0 (SPSS Inc., Chicago, IL, USA). The Shapiro-Wilk test was utilized to check the normality of the data. Based on the data’s distribution, group comparisons were conducted using the t-test, Mann-Whitney U test, Kruskal-Wallis test, or Chi-square test. Receiver Operating Characteristic (ROC) curves were employed to evaluate the area under the curve (AUC), determine optimal cut-off points, and establish the sensitivity and specificity for key parameters identified through logistic regression models. A p-value of less than 0.05 was considered to indicate statistical significance.

## Results

The study included a total of 81 patients diagnosed with iERM in the study group, with 95 cataract patients in the control group. The age and sex distributions were similar between the two groups (*p* = 0.513 and *p* = 0.194, respectively). Demographic data of both groups are presented in Table 1.

The median (min-max) values of NLR, PLR, SII, and SIRI in both groups are summarized in Table 2. In comparison to the control group, the iERM group had higher values of NLR (2.25 (1.09–12.71) vs. 1.91 (0.86–5.25), *p* = 0.003), PLR (117.22 (55.60-418.57) vs. 113.33 (34.38–312.50), *p* = 0.042), SII (529.45 (219.27-3725.29) vs. 472.57 (110.0-1312.50), *p* = 0.003), and SIRI (1.25 (0.48–6.28) vs. 0.90 (0.34–4.39), *p* < 0.001), respectively.

**Table 1:** Demographic data of participants

	iERMGroup (*n*:81)	Control Group (*n*:95)		*p*
Age, years (mean ± std)	67.91 ± 4.82	67.86 ± 7.60	0.513*	
Gender, Female Male	41 40	42 53	***0.194*** ^**^	

*iERM: Idiopathic epiretinal membrane; STD: Standard deviation*

*: Independent sample t-test;^**^: *Chi-square test*

**Table 2:** Comparison of blood-count-derived inflammatory markers

	iERM Group (*n*:81)	Control Group (*n*:95)	*p* ^*^
**NLR**, median (min-max)	2.25 (1.09–12.71)	1.91 (0.86–5.25)	*0.003*
**PLR**, median (min-max)	117.22 (55.60-418.57)	113.33 (34.38–312.50)	*0.042*
**SII**, median (min-max)	529.45 (219.27-3725.29)	472.52 (110.0-1312.50)	*0.003*
**SIRI**, median (min-max)	1.25 (0.48–6.28)	0.90 (0.34–4.39)	*< 0.001*

*iERM: Idiopathic epiretinal membrane; NLR: Neutrophil to lymphocyte ratio; PLR: Platelet to lymphocyte ratio; SII: Systemic immune-inflammation index; SIRI: Systemic inflammatory response index*

^*^: *Mann-Whitney U test*

**Fig. 2:**
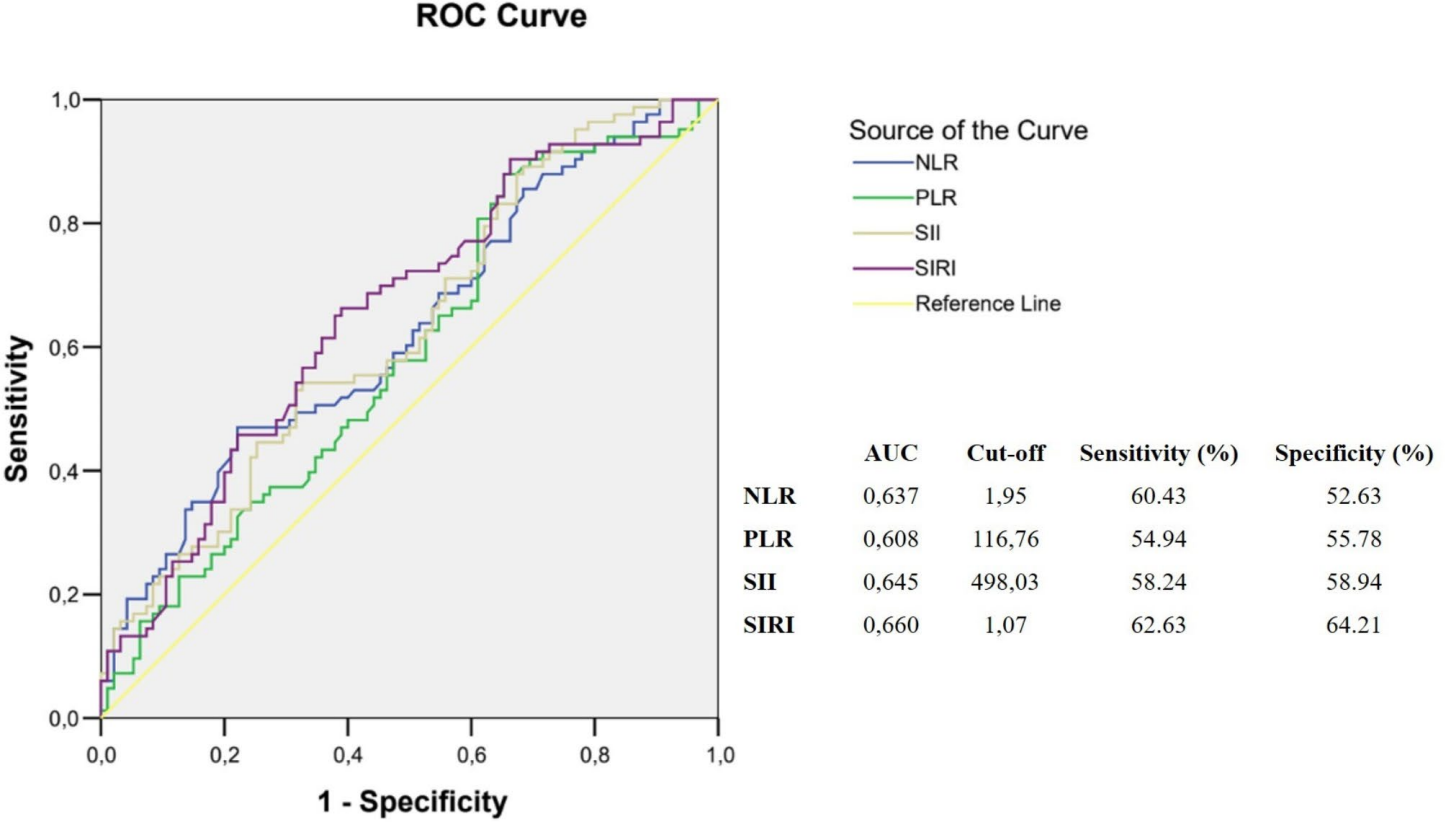
NLR, PLR, SII and SIRI receiver operating characteristic curves for iERM predictors *NLR: Neutrophil to lymphocyte ratio*,* unitless; PLR: Platelet to lymphocyte ratio*,* unitless; SII: Systemic immune-inflammation index*,* 10*^*9*^*/L; SIRI: Systemic inflammatory response index*,* 10*^*9*^*/L; iERM: Idiopathic epiretinal membrane*

According to ROC analysis, AUC values of NLR, PLR, SII, and SIRI were 0.637, 0.608, 0.645, and 0.660 for distinguishing iERM and control groups, respectively. The optimal cut-off values (with sensitivity and specificity) were determined as 1.95 (60.4% and 52.6%) for NLR, 116.76 (54.9% and 55.7%) for PLR, 498.03 (58.2% and 58.9%) for SII, and 1.07 (62.6% and 64.2%) for SIRI, respectively. (Fig. 2)

Table 3 shows subgroup analysis according to the iERM stages. No statistically significant differences were observed between groups in terms of NLR, PLR, SII, and SIRI (*p* = 0.244, *p* = 0.195, *p* = 0.248, and *p* = 0.465, respectively).

**Table 3:** Comparison of blood-count-derived inflammatory markers in different iERM satges

	Stage 1 (*n*:18)	Stage 2 (*n*:26)	Stage 3 (*n*:26)	Stage 4 (*n*:11)	*p* ^*^
**NLR**, Median (min-max)	2.21 (1.19–9.86)	1.95 (1.09–9.44)	2.39 (1.32–12.71)	2.48 (1.70–8.50)	*0.244*
**PLR**, median (min-max)	114.37 (55.60-258.57)	110.12 (56.25-301.88)	136.75 (78.64-418.57)	128.14 (69.29-236.67)	*0.195*
**SII**, median (min-max)	568.30 (235.71-1784.14)	462.10 (219.27-2247.78)	559.50 (275.23-3725.29)	624.84 (367.21-1708.50)	*0.248*
**SIRI**, median (min-max)	1.25 (0.60–5.91)	1.05 (0.48–6.28)	1.48 (0.55–2.57)	1.50 (0.51–5.95)	*0.465*

*iERM: Idiopathic epiretinal membrane; NLR: Neutrophil to lymphocyte ratio; PLR: Platelet to lymphocyte ratio; SII: Systemic immune-inflammation index; SIRI: Systemic inflammatory response index*

^*^: *Kruskal-Wallis test*

## Discussion

In this study, we evaluated the levels of blood-count-derived systemic inflammatory markers in patients with iERM compared to a control group. Our findings indicate that patients with iERM had significantly higher values of SII and SIRI, as well as elevated NLR and PLR, marking the first report of such increases in these specific markers in iERM patients. However, we found no significant differences in these inflammatory markers across different stages of iERM. These results suggest a potential link between systemic subclinical inflammation and the development of iERM, independent of its severity.

iERM is a prevalent condition affecting the vitreous-macular junction, especially in older adults with no apparent history of ocular disease [1, 2]. Although the exact mechanisms underlying its development are not entirely understood, previous studies have demonstrated the involvement of growth factors and cytokines in the formation of iERM [6–9]. Elevated cytokine and growth factor levels stimulate fibroblast activity and extracellular matrix contraction, leading to retinal thickening and folding. Müller cells are particularly influential in iERM formation, responding to mechanical stress and vitreal cytokines through hypertrophy, proliferation, and vascular leakage, which result in cellular contraction and retinal fold formation [5]. Histological analyses have also revealed the presence of myofibroblasts, fibroblasts, clear cells, and macrophages, all of which contribute to the formation and progression of iERM [16]. The involvement of glial cells, including Müller cells, astrocytes, and microglia, in reactive gliosis underscores the inflammatory nature of iERM, highlighting the intricate relationship between inflammation and cellular processes in its development.

Blood-count-derived inflammatory markers, NLR, PLR, SII, and SIRI, offer a convenient and cost-effective means of assessing the inflammatory status of patients in both systemic and ophthalmological diseases [10–13]. Specifically, in idiopathic epiretinal membrane (iERM), these markers can help identify underlying systemic inflammation contributing to the disease. Dikkaya et al. and Uzlu et al. revealed higher NLR in iERM patietns [17, 18]. Recently Demir et al. and Qin et al. reported higher levels of PLR in patients with iERM besides higher levels ol NLR [19, 20]. The present study is the first to document elevated SII and SIRI levels in patients with iERM, as well as elevated NLR and PLR. However, SIRI was found to have the higher sensivity (62.6%) and specificity (64.2%) than other biomarkers.

The lack of correlation between the grade of iERM and blood-count-derived inflammatory biomarkers underscores the complexity of ERM pathophysiology. While these biomarkers are indicative of systemic inflammation, they may not directly reflect the localized inflammatory processes occurring within the eye. ERM grading is based on the severity of retinal changes observed clinically, which may not correlate with systemic inflammatory markers due to the immune-privileged nature of the eye [21, 22]. Furthermore, ERM formation and progression involve multiple cellular and molecular mechanisms, including glial cell activation, extracellular matrix remodeling, and localized cytokine production, which may not be fully captured by systemic blood markers [4–9]. This disparity highlights the need for more specific intraocular biomarkers and advanced imaging techniques to better understand and assess the severity and progression of ERM, beyond what systemic inflammatory markers can reveal.

This study has several limitations. One major limitation is the retrospective design of the study which limited our ability to perform comprehensive analyses of various inflammatory markers beyond C-reactive protein and erythrocyte sedimentation rate in both the patient and control groups. Another constraint is the potential presence of iERM at the time of blood analyses, making it difficult to determine the predictive role of inflammatory parameters solely based on blood-count-derived biomarker calculations. Future research should include larger patient cohorts and investigate a broader range of inflammatory markers to better understand the systemic inflammation’s impact on iERM development. Moreover, comparing inflammatory infiltration in the histopathology of peeled ERM tissues with serum levels could provide further insights into the role of systemic inflammation in iERM.

In conclusion, the present study showed that patients with idiopathic epiretinal membrane (iERM) had elevated levels of NLR, PLR, SII, and SIRI compared to the control group. The latter marker has higher sensitivity and specificity. However, there was no correlation between these biomarkers and the severity of iERM. This suggests that subclinical systemic inflammation might play a role in the development of iERM, but not in determining how severe it becomes. To gain a clearer understanding of the connection between systemic inflammation and iERM development, more detailed and extensive research is required.

## Data Availability

The data that support the findings of this study are available from the corresponding author upon reasonable request.
